# Supplementary material for: Assessing the Relation between Plasma PCB Concentrations and Elevated Autistic Behaviours using Bayesian Predictive Odds Ratios
Source: Int J Environ Res Public Health. 2019 Feb 5;16(3):457. doi: 10.3390/ijerph16030457 (PMC6388164; doi:10.3390/ijerph16030457)
Supplement: Supplementary file 1 [file ijerph-16-00457-s001.pdf]

Supplementary materials for:

Assessing the Relation Between PCB Exposures During Pregnancy and Autistic Behaviour using Bayesian Predictive Odds Ratios

by Bernardo BA, Lanphear BP, Venners SA, Arbuckle TE, Braun JM, Muckle G, Fraser WD, and McCandless LC

Supplementary Figure 1. Scatterplots for Log2 Transformed Plasma PCB levels (quartiles) in relation to mean child SRS score in MIREC study participants. 1

Supplementary Table 1. Regression coefficients for the relation between participant covariates and mean SRS score in MIREC study participants, Canada, 2008-2011 using Multiple Linear Regression (n=546). 2

Supplementary Table 2. Plasma PCB levels (quartiles) in relation to Social Awareness score of MIREC study participants using Multiple Linear Regression (n=546). 4

Supplementary Table 3. Plasma PCB levels (quartiles) in relation to Social Cognition score of MIREC study participants using Multiple Linear Regression (n=546). 6

Supplementary Table 4. Plasma PCB levels (quartiles) in relation to Social Communication score of MIREC study participants using Multiple Linear Regression (n=546). 8

Supplementary Table 5. Plasma PCB levels (quartiles) in relation to Social Motivation score of MIREC study participants using Multiple Linear Regression (n=546). 10

Supplementary Table 6. Plasma PCB levels (quartiles) in relation to Restricted Interests and Repetitive Behaviour score of MIREC study participants using Multiple Linear Regression (n=546). 12

Supplementary Table 7. Plasma PCB levels (quartiles) in relation to SRS score of MIREC study participants with male babies using Multiple Linear Regression (n=261). 14

Supplementary Table 8. Plasma PCB levels (quartiles) in relation to SRS score of MIREC study participants with female babies using Multiple Linear Regression (n=285). 16

Supplementary Table 9. Log2 Transformed Plasma PCB levels (quartiles) in relation to mean child SRS score in MIREC study participants, Canada, 2008-2011 using Multiple Linear Regression (n=546). 18

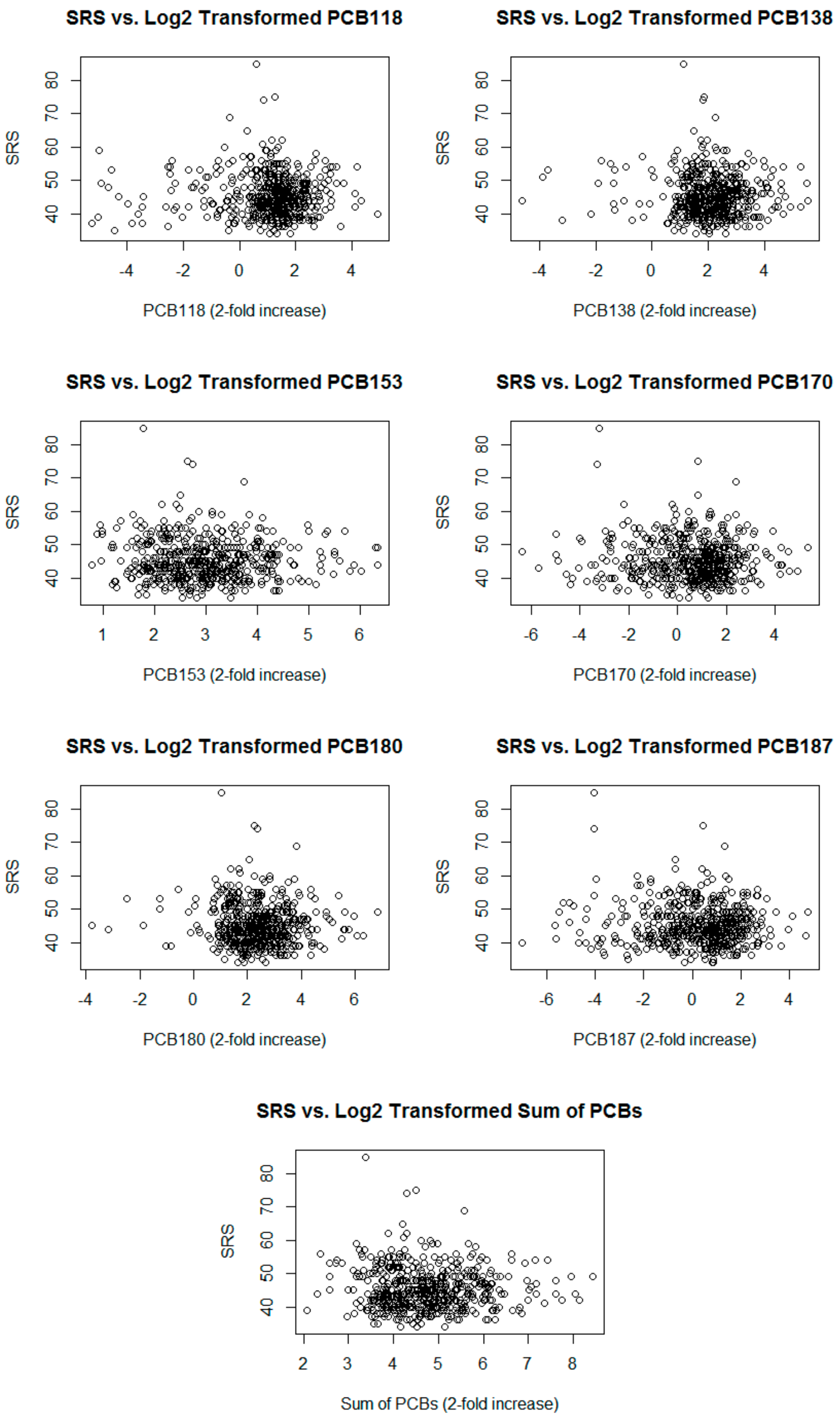

Supplementary Figure 1. Scatterplots for Log2 Transformed Plasma PCB levels (quartiles) in relation to mean child SRS score in MIREC study participants.

Supplementary Table 1. Regression coefficients for the relation between participant covariates and mean SRS score in MIREC study participants, Canada, 2008-2011 using Multiple Linear Regression (n=546).

	n (%)	SRS Unadjusted mean scores (95% CI)	SRS Adjusted[1] mean scores (95% CI)
**Child Sex**			
Intercept		44.2 (43.5, 44.9)	45.5 (41.7, 49.4)
Female	285 (52.2)	0.0 (referent)	0.0
Male	261 (47.8)	2.4 (1.4, 3.4)	2.3 (1.3, 3.3)
**Mother's Age**			
Intercept		46.7 (45.6, 47.8)	45.5 (41.7, 49.4)
19-29	122 (22.3)	0.0	0.0
30-34	205 (37.5)	-1.3 (-2.7, 0.0)	-0.2 (-1.6, 1.2)
35+	219 (40.0)	-2.2 (-3.5, -0.8)	-1.1 (-2.5, 0.3)
**Race**			
Intercept		45.2 (44.6, 45.7)	45.5 (41.7, 49.4)
White	491 (89.9)	0.0	0.0
Other	55 (10.1)	1.7 (0.0, 3.4)	1.4 (-0.4, 3.1)
**Marital Status**			
Intercept		44.7 (44.1, 45.3)	45.5 (41.7, 49.4)
Married	241 (89.9)	0.0	0.0
Other	154 (28.2)	2.2 (1.1, 3.3)	1.5 (0.3, 2.7)
**Education Level**			
Intercept		47.8 (45.6, 50.0)	45.5 (41.7, 49.4)
High School Diploma or less	29 (5.3)	0.0	0.0
College or Trade School Diploma	154 (28.2)	-1.6 (-4.0, 0.8)	-0.9 (-3.3, 1.5)
Undergraduate University Degree	213 (39.0)	-2.3 (-4.7, 0.0)	-1.1 (-3.5, 1.3)
Graduate University Degree	150 (27.5)	-4.0 (-6.4, -1.6)	-2.3 (-4.8, 0.2)
**Annual Income**			

Intercept		47.2 (45.8, 48.6)	45.5 (41.7, 49.4)
<= $40,000	73 (13.4)	0.0	0.0
$40,001-$80,000	151 (27.7)	-1.0 (-2.7, 0.7)	-0.3 (-2.1, 1.4)
$80,001-$100,000	105 (19.2)	-2.1 (-3.9, -0.3)	-1.1 (-3.0, 0.7)
> $100,000	217 (39.7)	-3.0 (-4.6, -1.4)	-1.5 (-3.3, 0.3)
**Has Ever Smoked During Pregnancy**			
Intercept		45.2 (44.5, 44.5)	45.5 (41.7, 49.4)
No	357 (65.4)	0.0	0.0
Yes	189 (34.6)	0.4 (-0.6, 1.5)	0.3 (-0.8, 1.4)
**Has Ever Consumed Alcohol During Pregnancy**			
Intercept		45.4 (44.9, 46.0)	45.5 (41.7, 49.4)
No	455 (83.3)	0.0	0.0
Yes	91 (16.7)	-0.7 (-2.1, 0.7)	-0.8 (-2.2, 0.6)
**Pre-Pregnancy BMI**			
Intercept		45.2 (42, 48.5)	45.5 (41.7, 49.4)
Underweight	14 (2.6)	0.0	0.0
Normal	332 (60.8)	0.2 (-3.1, 3.5)	1.2 (-2.1, 4.4)
Overweight	112 (20.5)	-0.5 (-3.9, 2.9)	-0.1 (-3.4, 3.3)
Obese	88 (16.1)	0.7 (-2.8, 4.2)	0.9 (-2.6, 4.3)

[1] Adjusted for child's sex, mother's age, race, marital status, education level, annual income, whether the mother has ever smoked during pregnancy, has ever consumed alcohol during pregnancy, and pre-pregnancy BMI.

Supplementary Table 2. Plasma PCB levels (quartiles) in relation to Social Awareness score of MIREC study participants using Multiple Linear Regression (n=546).

PCB Category	Value (ng/g lipid)	n	SRS Unadjusted mean scores (95% CI)	SRS Adjusted* mean scores (95% CI)
**PCB118**				
Q1	< 1.4	108	0.0 (referent)	0.0
Q2	1.4-< 2.3	143	-1.83 (-3.33, -0.33)	-1.75 (-3.74, 0.23)
Q3	2.3-< 3.6	170	-1.65 (-3.08, -0.22)	-1.25 (-3.19, 0.70)
Q4	>= 3.6	125	-2.30 (-3.87, -0.73)	-1.76 (-3.84, 0.33)
**PCB138**				
Q1	< 3.2	175	0.0	0.0
Q2	3.2-< 5.5	184	-1.22 (-2.61, 0.17)	-0.80 (-2.52, 0.92)
Q3	5.5-< 8.9	118	-0.04 (-1.65, 1.57)	0.47 (-1.51, 2.45)
Q4	>= 8.9	69	-1.34 (-3.34, 0.66)	-0.69 (-3.01, 1.62)
**PCB153**				
Q1	< 4.2	87	0.0	0.0
Q2	4.2-< 7.4	178	-0.83 (-2.24, 0.58)	-0.78 (-2.85, 1.28)
Q3	7.4-< 11.7	144	-1.99 (-3.49, -0.49)	-1.65 (-3.93, 0.64)
Q4	>= 11.7	137	-0.93 (-2.45, 0.59)	-0.27 (-2.60, 2.05)
**PCB170**				
Q1	< 1.5	227	0.0	0.0
Q2	1.5-< 2.6	141	-0.47 (-1.98, 1.04)	0.12 (-1.60, 1.85)
Q3	2.6-< 4.3	110	-1.45 (-3.10, 0.20)	-0.43 (-2.36, 1.51)
Q4	>= 4.3	68	-0.30 (-2.31, 1.71)	0.67 (-1.63, 2.96)
**PCB180**				
Q1	< 3.4	154	0.0	0.0
Q2	3.4-< 6.1	182	-3.08 (-4.47, -1.69)	-2.93 (-4.76, -1.09)
Q3	6.1-< 10.4	120	-2.44 (-4.04, -0.84)	-2.03 (-4.15, 0.10)

Q4	>= 10.4	90	-1.65 (-3.44, 0.14)	-1.17 (-3.49, 1.15)
**PCB187**				
Q1	< 0.92	197	0.0	0.0
Q2	0.92-< 1.8	124	-0.03 (-1.61, 1.55)	-0.19 (-1.94, 1.56)
Q3	1.8-< 3.3	135	-0.63 (-2.16, 0.90)	-0.22 (-2.03, 1.59)
Q4	>= 3.3	90	-0.55 (-2.34, 1.24)	0.34 (-1.76, 2.44)
**Sum of above PCBs**				
Q1	< 33.4	358	0.0	0.0
Q2	33.4-< 55.3	110	-0.16 (-1.81, 1.49)	0.68 (-1.08, 2.43)
Q3	55.3-< 86.3	51	-0.40 (-2.70, 1.90)	0.31 (-2.10, 2.72)
Q4	>= 86.3	27	-0.92 (-4.06, 2.22)	-0.30 (-3.46, 2.87)

* Adjusted for child's sex, mother's age, race, marital status, education level, annual income, whether the mother has ever smoked during pregnancy, has ever consumed alcohol during pregnancy, and pre-pregnancy BMI.

Supplementary Table 3. Plasma PCB levels (quartiles) in relation to Social Cognition score of MIREC study participants using Multiple Linear Regression (n=546).

PCB Category	Value (ng/g lipid)	n	SRS Unadjusted mean scores (95% CI)	SRS Adjusted* mean scores (95% CI)
**PCB118**				
Q1	< 1.4	108	0.0 (referent)	0.0
Q2	1.4-< 2.3	143	-0.17 (-3.03, -0.63)	-0.16 (-1.75, 1.42)
Q3	2.3-< 3.6	170	-0.85 (-2.79, -0.51)	-0.49 (-2.05, 1.07)
Q4	>= 3.6	125	-0.72 (-3.56, -1.04)	-0.22 (-1.88, 1.45)
**PCB138**				
Q1	< 3.2	175	0.0	0.0
Q2	3.2-< 5.5	184	-0.74 (-2.34, -0.10)	-0.09 (-1.46, 1.28)
Q3	5.5-< 8.9	118	-1.16 (-1.32, 1.24)	-0.52 (-2.10, 1.06)
Q4	>= 8.9	69	0.06 (-2.94, 0.26)	0.72 (-1.12, 2.57)
**PCB153**				
Q1	< 4.2	87	0.0	0.0
Q2	4.2-< 7.4	178	-0.74 (-1.96, 0.30)	-0.48 (-2.12, 1.15)
Q3	7.4-< 11.7	144	-2.57 (-3.18, -0.80)	-2.06 (-3.87, -0.26)
Q4	>= 11.7	137	-0.89 (-2.15, 0.29)	-0.09 (-1.93, 1.75)
**PCB170**				
Q1	< 1.5	227	0.0	0.0
Q2	1.5-< 2.6	141	-0.39 (-1.68, 0.74)	0.10 (-1.28, 1.47)
Q3	2.6-< 4.3	110	-1.13 (-2.77, -0.13)	-0.20 (-1.74, 1.34)
Q4	>= 4.3	68	-0.57 (-1.91, 1.31)	0.21 (-1.62, 2.04)
**PCB180**				
Q1	< 3.4	154	0.0	0.0
Q2	3.4-< 6.1	182	-2.06 (-4.20, -1.96)	-1.41 (-2.87, 0.06)
Q3	6.1-< 10.4	120	-2.42 (-3.71, -1.17)	-1.47 (-3.18, 0.23)

Q4	>= 10.4	90	-1.13 (-3.08, -0.22)	-0.48 (-2.33, 1.38)
**PCB187**				
Q1	< 0.92	197	0.0	0.0
Q2	0.92-< 1.8	124	-0.37 (-1.29, 1.23)	-0.55 (-1.94, 0.84)
Q3	1.8-< 3.3	135	-0.92 (-1.85, 0.59)	-0.55 (-1.99, 0.89)
Q4	>= 3.3	90	-0.53 (-1.98, 0.88)	0.04 (-1.64, 1.71)
**Sum of above PCBs**				
Q1	< 33.4	358	0.0	0.0
Q2	33.4-< 55.3	110	-0.79 (-1.48, 1.16)	0.06 (-1.34, 1.46)
Q3	55.3-< 86.3	51	-0.03 (-2.24, 1.44)	0.22 (-1.70, 2.14)
Q4	>= 86.3	27	0.47 (-3.43, 1.59)	1.14 (-1.38, 3.66)

* Adjusted for child's sex, mother's age, race, marital status, education level, annual income, whether the mother has ever smoked during pregnancy, has ever consumed alcohol during pregnancy, and pre-pregnancy BMI.

Supplementary Table 4. Plasma PCB levels (quartiles) in relation to Social Communication score of MIREC study participants using Multiple Linear Regression (n=546).

PCB Category	Value (ng/g lipid)	n	SRS Unadjusted mean scores (95% CI)	SRS Adjusted* mean scores (95% CI)
**PCB118**				
Q1	< 1.4	108	0.0 (referent)	0.0
Q2	1.4-< 2.3	143	-0.21 (-3.04, -0.62)	0.08 (-1.49, 1.64)
Q3	2.3-< 3.6	170	-0.31 (-2.80, -0.50)	0.36 (-1.18, 1.89)
Q4	>= 3.6	125	-0.11 (-3.56, -1.04)	0.71 (-0.93, 2.35)
**PCB138**				
Q1	< 3.2	175	0.0	0.0
Q2	3.2-< 5.5	184	0.10 (-2.34, -0.10)	0.76 (-0.59, 2.11)
Q3	5.5-< 8.9	118	-0.05 (-1.33, 1.25)	0.72 (-0.84, 2.27)
Q4	>= 8.9	69	0.81 (-2.95, 0.27)	1.76 (-0.06, 3.57)
**PCB153**				
Q1	< 4.2	87	0.0	0.0
Q2	4.2-< 7.4	178	-0.22 (-1.96, 0.30)	0.10 (-1.52, 1.72)
Q3	7.4-< 11.7	144	-1.11 (-3.19, -0.79)	-0.31 (-2.10, 1.48)
Q4	>= 11.7	137	0.15 (-2.15, 0.29)	1.34 (-0.49, 3.16)
**PCB170**				
Q1	< 1.5	227	0.0	0.0
Q2	1.5-< 2.6	141	-1.06 (-1.68, 0.74)	-0.46 (-1.82, 0.89)
Q3	2.6-< 4.3	110	-0.78 (-2.78, -0.12)	0.33 (-1.19, 1.84)
Q4	>= 4.3	68	0.08 (-1.92, 1.32)	1.05 (-0.76, 2.85)
**PCB180**				
Q1	< 3.4	154	0.0	0.0
Q2	3.4-< 6.1	182	-2.33 (-4.20, -1.96)	-1.77 (-3.21, -0.33)
Q3	6.1-< 10.4	120	-1.51 (-3.72, -1.16)	-0.42 (-2.09, 1.25)

Q4	>= 10.4	90	-0.42 (-3.08, -0.22)	0.47 (-1.35, 2.29)
**PCB187**				
Q1	< 0.92	197	0.0	0.0
Q2	0.92-< 1.8	124	-0.35 (-1.30, 1.24)	-0.48 (-1.86, 0.89)
Q3	1.8-< 3.3	135	-0.49 (-1.86, 0.60)	0.04 (-1.38, 1.46)
Q4	>= 3.3	90	-0.17 (-1.99, 0.89)	0.70 (-0.95, 2.36)
**Sum of above PCBs**				
Q1	< 33.4	358	0.0	0.0
Q2	33.4-< 55.3	110	-0.03 (-1.49, 1.17)	0.99 (-0.38, 2.37)
Q3	55.3-< 86.3	51	0.32 (-2.25, 1.45)	0.88 (-1.01, 2.78)
Q4	>= 86.3	27	0.86 (-3.44, 1.60)	1.67 (-0.81, 4.15)

* Adjusted for child's sex, mother's age, race, marital status, education level, annual income, whether the mother has ever smoked during pregnancy, has ever consumed alcohol during pregnancy, and pre-pregnancy BMI.

Supplementary Table 5. Plasma PCB levels (quartiles) in relation to Social Motivation score of MIREC study participants using Multiple Linear Regression (n=546).

**PCB Category**	**Value (ng/g lipid)**	**n**	**SRS Unadjusted mean scores (95% CI)**	**SRS Adjusted* mean scores (95% CI)**
**PCB118**				
Q1	< 1.4	108	0.0 (referent)	0.0
Q2	1.4-< 2.3	143	-0.16 (-3.24, -0.42)	0.03 (-1.84, 1.90)
Q3	2.3-< 3.6	170	-1.02 (-2.99, -0.31)	-0.52 (-2.36, 1.32)
Q4	>= 3.6	125	-0.62 (-3.78, -0.82)	0.01 (-1.95, 1.98)
**PCB138**				
Q1	< 3.2	175	0.0	0.0
Q2	3.2-< 5.5	184	-0.33 (-2.53, 0.09)	0.03 (-1.59, 1.65)
Q3	5.5-< 8.9	118	-0.73 (-1.55, 1.47)	-0.38 (-2.25, 1.49)
Q4	>= 8.9	69	-0.42 (-3.22, 0.54)	0.01 (-2.17, 2.19)
**PCB153**				
Q1	< 4.2	87	0.0	0.0
Q2	4.2-< 7.4	178	-0.15 (-2.15, 0.49)	-0.12 (-2.07, 1.83)
Q3	7.4-< 11.7	144	-1.25 (-3.4, -0.58)	-0.96 (-3.11, 1.19)
Q4	>= 11.7	137	-1.04 (-2.36, 0.50)	-0.58 (-2.77, 1.62)
**PCB170**				
Q1	< 1.5	227	0.0	0.0
Q2	1.5-< 2.6	141	-1.70 (-1.88, 0.94)	-1.72 (-3.34, -0.10)
Q3	2.6-< 4.3	110	-1.55 (-3.00, 0.10)	-1.19 (-3.01, 0.62)
Q4	>= 4.3	68	-0.66 (-2.19, 1.59)	-0.61 (-2.76, 1.55)
**PCB180**				
Q1	< 3.4	154	0.0	0.0
Q2	3.4-< 6.1	182	-1.08 (-4.40, -1.76)	-0.96 (-2.69, 0.78)
Q3	6.1-< 10.4	120	-2.14 (-3.93, -0.95)	-1.62 (-3.64, 0.39)

Q4	>= 10.4	90	-0.27 (-3.33, 0.03)	-0.18 (-2.38, 2.02)
**PCB187**				
Q1	< 0.92	197	0.0	0.0
Q2	0.92-< 1.8	124	-1.39 (-1.51, 1.45)	-1.44 (-3.08, 0.19)
Q3	1.8-< 3.3	135	-2.35 (-2.06, 0.80)	-2.22 (-3.91, -0.53)
Q4	>= 3.3	90	-0.69 (-2.23, 1.13)	-0.45 (-2.41, 1.51)
**Sum of above PCBs**				
Q1	< 33.4	358	0.0	0.0
Q2	33.4-< 55.3	110	-1.29 (-1.71, 1.39)	-0.72 (-2.37, 0.93)
Q3	55.3-< 86.3	51	-0.11 (-2.56, 1.76)	-0.09 (-2.36, 2.18)
Q4	>= 86.3	27	-0.33 (-3.87, 2.03)	0.09 (-2.89, 3.06)

* Adjusted for child's sex, mother's age, race, marital status, education level, annual income, whether the mother has ever smoked during pregnancy, has ever consumed alcohol during pregnancy, and pre-pregnancy BMI.

Supplementary Table 6. Plasma PCB levels (quartiles) in relation to Restricted Interests and Repetitive Behaviour score of MIREC study participants using Multiple Linear Regression (n=546).

PCB Category	Value (ng/g lipid)	n	SRS Unadjusted mean scores (95% CI)	SRS Adjusted* mean scores (95% CI)
**PCB118**				
Q1	< 1.4	108	0.0 (referent)	0.0
Q2	1.4-< 2.3	143	-0.64 (-3.17, -0.49)	-0.39 (-2.15, 1.38)
Q3	2.3-< 3.6	170	-0.34 (-2.92, -0.38)	0.16 (-1.57, 1.89)
Q4	>= 3.6	125	-0.45 (-3.71, -0.89)	0.23 (-1.62, 2.08)
**PCB138**				
Q1	< 3.2	175	0.0	0.0
Q2	3.2-< 5.5	184	0.42 (-2.47, 0.03)	1.12 (-0.40, 2.64)
Q3	5.5-< 8.9	118	-0.26 (-1.48, 1.40)	0.32 (-1.43, 2.07)
Q4	>= 8.9	69	1.13 (-3.13, 0.45)	1.91 (-0.13, 3.96)
**PCB153**				
Q1	< 4.2	87	0.0	0.0
Q2	4.2-< 7.4	178	0.50 (-2.09, 0.43)	0.93 (-0.89, 2.76)
Q3	7.4-< 11.7	144	-1.20 (-3.32, -0.66)	-0.53 (-2.55, 1.48)
Q4	>= 11.7	137	0.41 (-2.29, 0.43)	1.34 (-0.72, 3.39)
**PCB170**				
Q1	< 1.5	227	0.0	0.0
Q2	1.5-< 2.6	141	-1.24 (-1.82, 0.88)	-0.83 (-2.36, 0.69)
Q3	2.6-< 4.3	110	-1.39 (-2.92, 0.02)	-0.60 (-2.31, 1.10)
Q4	>= 4.3	68	0.60 (-2.10, 1.50)	1.27 (-0.76, 3.29)
**PCB180**				
Q1	< 3.4	154	0.0	0.0
Q2	3.4-< 6.1	182	-2.03 (-4.33, -1.83)	-1.61 (-3.23, 0.02)
Q3	6.1-< 10.4	120	-2.17 (-3.86, -1.02)	-1.51 (-3.40, 0.38)

Q4	>= 10.4	90	-0.12 (-3.24, -0.06)	0.46 (-1.59, 2.52)
**PCB187**				
Q1	< 0.92	197	0.0	0.0
Q2	0.92-< 1.8	124	-0.93 (-1.44, 1.38)	-1.15 (-2.70, 0.39)
Q3	1.8-< 3.3	135	-0.74 (-2.00, 0.74)	-0.44 (-2.04, 1.16)
Q4	>= 3.3	90	-0.13 (-2.15, 1.05)	0.48 (-1.38, 2.33)
**Sum of above PCBs**				
Q1	< 33.4	358	0.0	0.0
Q2	33.4-< 55.3	110	-0.78 (-1.63, 1.31)	-0.05 (-1.60, 1.50)
Q3	55.3-< 86.3	51	0.13 (-2.45, 1.65)	0.43 (-1.70, 2.56)
Q4	>= 86.3	27	1.81 (-3.72, 1.88)	2.46 (-0.33, 5.26)

* Adjusted for child’s sex, mother’s age, race, marital status, education level, annual income, whether the mother has ever smoked during pregnancy, has ever consumed alcohol during pregnancy, and pre-pregnancy BMI.

Supplementary Table 7. Plasma PCB levels (quartiles) in relation to SRS score of MIREC study participants with male babies using Multiple Linear Regression (n=261).

PCB Category	Value (ng/g lipid)	n	SRS Unadjusted mean scores (95% CI)	SRS Adjusted* mean scores (95% CI)
**PCB118**				
Q1	< 1.4	55	0.0 (referent)	0.0
Q2	1.4-< 2.3	63	1.05 (-0.86, 2.96)	1.44 (-1.20, 4.08)
Q3	2.3-< 3.6	82	-0.11 (-1.87, 1.65)	0.85 (-1.63, 3.33)
Q4	>= 3.6	61	-0.63 (-2.55, 1.29)	0.42 (-2.16, 3.01)
**PCB138**				
Q1	< 3.2	77	0.0	0.0
Q2	3.2-< 5.5	90	-0.49 (-2.21, 1.23)	0.03 (-2.23, 2.29)
Q3	5.5-< 8.9	61	-0.65 (-2.59, 1.29)	0.11 (-2.42, 2.64)
Q4	>= 8.9	33	0.70 (-1.75, 3.15)	1.78 (-1.21, 4.76)
**PCB153**				
Q1	< 4.2	37	0.0	0.0
Q2	4.2-< 7.4	82	-0.72 (-2.48, 1.04)	-0.40 (-3.17, 2.36)
Q3	7.4-< 11.7	79	-3.01 (-4.76, -1.26)	-2.26 (-5.24, 0.73)
Q4	>= 11.7	63	-0.42 (-2.32, 1.48)	0.56 (-2.52, 3.65)
**PCB170**				
Q1	< 1.5	109	0.0	0.0
Q2	1.5-< 2.6	72	-3.47 (-5.27, -1.67)	-2.79 (-4.90, -0.68)
Q3	2.6-< 4.3	50	-2.22 (-4.30, -0.14)	-1.76 (-4.20, 0.68)
Q4	>= 4.3	30	0.01 (-2.54, 2.56)	1.19 (-1.84, 4.22)
**PCB180**				
Q1	< 3.4	74	0.0	0.0
Q2	3.4-< 6.1	89	-3.62 (-5.32, -1.92)	-3.54 (-5.83, -1.26)
Q3	6.1-< 10.4	59	-3.19 (-5.14, -1.24)	-2.90 (-5.57, -0.22)

Q4	>= 10.4	39	-0.33 (-2.60, 1.94)	0.09 (-2.90, 3.08)
**PCB187**				
Q1	< 0.92	90	0.0	0.0
Q2	0.92-< 1.8	64	-0.03 (-1.94, 1.88)	-0.37 (-2.56, 1.82)
Q3	1.8-< 3.3	68	-0.77 (-2.63, 1.09)	-0.46 (-2.78, 1.86)
Q4	>= 3.3	39	0.11 (-2.18, 2.40)	0.88 (-1.87, 3.63)
**Sum of above PCBs**				
Q1	< 33.4	172	0.0	0.0
Q2	33.4-< 55.3	54	-0.25 (-2.28, 1.78)	0.81 (-1.39, 3.00)
Q3	55.3-< 86.3	24	-0.31 (-3.18, 2.56)	0.18 (-2.95, 3.31)
Q4	>= 86.3	11	1.65 (-2.40, 5.70)	2.94 (-1.25, 7.13)

* Adjusted for child's sex, mother's age, race, marital status, education level, annual income, whether the mother has ever smoked during pregnancy, has ever consumed alcohol during pregnancy, and pre-pregnancy BMI.

Supplementary Table 8. Plasma PCB levels (quartiles) in relation to SRS score of MIREC study participants with female babies using Multiple Linear Regression (n=285).

PCB Category	Value (ng/g lipid)	n	SRS Unadjusted mean scores (95% CI)	SRS Adjusted* mean scores (95% CI)
**PCB118**				
Q1	< 1.4	52	0.0 (referent)	0.0
Q2	1.4-< 2.3	81	-1.62 (-3.05, -0.19)	-1.56 (-3.46, 0.34)
Q3	2.3-< 3.6	89	-1.47 (-2.86, -0.08)	-0.84 (-2.74, 1.05)
Q4	>= 3.6	63	-0.77 (-2.32, 0.78)	0.00 (-2.07, 2.06)
**PCB138**				
Q1	< 3.2	97	0.0	0.0
Q2	3.2-< 5.5	95	-0.37 (-1.73, 0.99)	0.55 (-1.10, 2.20)
Q3	5.5-< 8.9	58	-0.67 (-2.28, 0.94)	0.42 (-1.52, 2.37)
Q4	>= 8.9	35	-0.38 (-2.35, 1.59)	0.63 (-1.58, 2.84)
**PCB153**				
Q1	< 4.2	49	0.0	0.0
Q2	4.2-< 7.4	97	-0.09 (-1.45, 1.27)	0.39 (-1.55, 2.32)
Q3	7.4-< 11.7	66	-0.94 (-2.47, 0.59)	0.37 (-1.83, 2.57)
Q4	>= 11.7	73	-0.59 (-2.06, 0.88)	1.01 (-1.18, 3.20)
**PCB170**				
Q1	< 1.5	117	0.0	0.0
Q2	1.5-< 2.6	70	1.15 (-0.34, 2.64)	1.75 (0.05, 3.46)
Q3	2.6-< 4.3	61	-0.29 (-1.86, 1.28)	1.24 (-0.64, 3.12)
Q4	>= 4.3	37	-0.03 (-1.95, 1.89)	1.23 (-0.90, 3.37)
**PCB180**				
Q1	< 3.4	79	0.0	0.0
Q2	3.4-< 6.1	94	-1.15 (-2.52, 0.22)	-0.18 (-1.98, 1.62)
Q3	6.1-< 10.4	62	-1.39 (-2.95, 0.17)	0.31 (-1.80, 2.43)

Q4	>= 10.4	50	-0.75 (-2.44, 0.94)	0.57 (-1.65, 2.79)
**PCB187**				
Q1	< 0.92	106	0.0	0.0
Q2	0.92-< 1.8	61	-1.54 (-3.11, 0.03)	-1.05 (-2.78, 0.69)
Q3	1.8-< 3.3	68	-1.51 (-3.02, 0.00)	-0.38 (-2.15, 1.40)
Q4	>= 3.3	50	-0.76 (-2.45, 0.93)	0.43 (-1.56, 2.42)
**Sum of above PCBs**				
Q1	< 33.4	185	0.0	0.0
Q2	33.4-< 55.3	57	-0.96 (-2.57, 0.65)	0.00 (-1.73, 1.74)
Q3	55.3-< 86.3	28	0.46 (-1.75, 2.67)	0.90 (-1.39, 3.20)
Q4	>= 86.3	15	0.05 (-2.90, 3.00)	0.95 (-2.03, 3.92)

* Adjusted for child's sex, mother's age, race, marital status, education level, annual income, whether the mother has ever smoked during pregnancy, has ever consumed alcohol during pregnancy, and pre-pregnancy BMI.

Supplementary Table 9. Log2 Transformed Plasma PCB levels (quartiles) in relation to mean child SRS score in MIREC study participants, Canada, 2008-2011 using Multiple Linear Regression (n=546).

PCB (2-fold increase)	SRS Unadjusted mean scores (95% CI)	SRS Adjusted* mean scores (95% CI)
**PCB118**	-0.07 (-0.44, 0.30)	0.09 (-0.28, 0.46)
**PCB138**	-0.17 (-0.63, 0.28)	0.14 (-0.34, 0.61)
**PCB153**	-0.23 (-0.77, 0.32)	0.16 (-0.44, 0.75)
**PCB170**	-0.19 (-0.49, 0.12)	0.04 (-0.28, 0.37)
**PCB180**	-0.28 (-0.72, 0.16)	0.10 (-0.40, 0.60)
**PCB187**	-0.16 (-0.46, 0.14)	-0.04 (-0.36, 0.27)
**Sum of above PCBs**	-0.25 (-0.79, 0.28)	0.15 (-0.43, 0.73)

* Adjusted for child's sex, mother's age, race, marital status, education level, annual income, whether the mother has ever smoked during pregnancy, has ever consumed alcohol during pregnancy, and pre-pregnancy BMI.
